# Treatment of cognitive and mood disorders secondary to traumatic brain injury by the association of bilateral occipital nerve stimulation and a combined protocol of multisite repetitive transcranial magnetic stimulation and cognitive training: A case report

**DOI:** 10.3389/fneur.2023.1195513

**Published:** 2023-11-07

**Authors:** Tiphanie Caloc'h, Estelle Le Saout, Séverine Litaneur, Alcira Suarez, Sylvain Durand, Jean-Pascal Lefaucheur, Jean-Paul Nguyen

**Affiliations:** ^1^Unité de stimulation transcrânienne, Clinique Bretéché, Groupe Elsan, Nantes, France; ^2^EA 4391, équipe ENT (Excitabilité Nerveuse et Thérapeutique), Université Paris-Est Créteil, Créteil, France; ^3^Unité de Neurophysiologie Clinique, Hôpital Henri Mondor, Assistance Publique - Hôpitaux de Paris, Créteil, France

**Keywords:** traumatic brain injury, refractory headache, occipital nerve stimulation, repetitive transcranial magnetic simulation, cognitive training, combined strategy

## Abstract

**Purpose:**

Cognitive impairment secondary to traumatic brain injury (TBI) is difficult to treat and usually results in severe disability.

**Method:**

A 48-year-old man presented with chronic refractory headaches and persistent disabling cognitive impairment after TBI. He was first treated with occipital nerve stimulation (ONS) implanted bilaterally to relieve headaches (8 years after the head trauma). Two years later, he was treated with a 6-week protocol combining repetitive transcranial magnetic stimulation (rTMS) delivered to multiple cortical sites (prefrontal cortex, language areas, and areas involved in visuo-spatial functions) and computerized cognitive training (CogT) (targeting memory, language, and visuo-spatial functions) to improve cognitive performance.

**Results:**

Executive and cognitive functions (attention, ability to perform calculations, and verbal fluency) improved in association with pain relief after ONS (33–42% improvement) and then improved even more after the rTMS-CogT protocol with an additional improvement of 36–40% on apathy, depression, and anxiety, leading to a significant reduction in caregiver burden. The functional improvement persisted and even increased at 6 months after the end of the rTMS-CogT procedure (10 years after the onset of TBI and 2 years after ONS implantation).

**Conclusion:**

This is the first observation describing sustained improvement in post-TBI refractory headache, depression, and cognitive impairment by the association of bilaterally implanted ONS and a combined procedure of multisite rTMS and CogT to target various brain functions.

## Introduction

Cognitive impairment is one of the most common sequelae of traumatic brain injury (TBI) (1). Their number tends to increase with the increase in the number of survivors, linked to better management of severe head injuries in the acute phase (2). Cognitive sequelae are considered more disabling than motor sequelae (3–7). They play an important role in hindering the possibility of reintegration into working life. The most frequent disorders concern memory (8), attention (9), and executive functions (10). Classic treatment consists of cognitive rehabilitation combined with drug treatments used in the cognitive disorders of Alzheimer's disease, such as cholinesterase inhibitors (Donezepil, Rivastigmine) (11). However, these treatments have side effects and only act inconsistently (12).

The efficacy of cognitive rehabilitation depends above all on the patient's participation, which may be disrupted by stress, a depressive state or other symptoms encountered in the context of post-TBI syndrome, such as headaches. These different factors must be taken into account before starting cognitive rehabilitation (11).

Recovery from a cognitive deficit can also be hampered by other factors, such as arousal and attention disorders. These disorders are common when trauma causes shear damage to the white matter responsible for a disconnection between the thalamus and the neocortex (13–15). Thus, interventions that can stimulate the thalamus directly [thalamic stimulation (16)] or indirectly [stimulation of the vagus nerve or peripheral nerves (17)] have been proposed in this context.

Currently, it is considered that the goal of cognitive rehabilitation is to develop neuroplasticity that will make the neural circuits involved in cognition more efficient (18). The outcome could be optimized by combining cognitive rehabilitation with a technique of non-invasive stimulation of the cerebral cortex such as repetitive transcranial magnetic stimulation (rTMS) or transcranial direct current stimulation (tDCS) (19), which are known to promote synaptic plasticity.

We report the case of a patient who suffered a head injury responsible for a post-TBI syndrome with disabling headaches and cognitive disorders. A first treatment with surgically implanted occipital nerve stimulation (ONS) was effective on headaches and clearly led to a cognitive improvement. Subsequently, a treatment combining multisite rTMS with computerized cognitive training (CogT) (20–22) further improved cognitive performance and acted on depression, anxiety, and apathy.

## Case report

This is a 48-year-old patient who in 2012 had severe head injury related to a serious quad bike accident. On the initial CT scan, there was a fracture of the temporal and petrous bones on the left side, a diffuse subarachnoid hemorrhage and an edematous parenchymal contusion predominating on the right side, accompanied by a right temporal subdural hematoma (3 mm thick). A follow-up CT scan performed 72 h later showed a small intracerebral hemorrhage localized in the right temporal region, with increased edema in the right temporoparietal region leading to a mass effect with displacement of midline structures and the effacement of some cortical sulci, but without signs of cerebral herniation. The patient initially presented with an intermediate disorder of consciousness (Glasgow score 12) which lasted 5 days. Afterwards, the patient rapidly complained of headaches, followed by memory and attention disturbances and verbal expression difficulties. He also became irritable and had sleep disorders with difficulty falling asleep and frequent waking up. Daily headaches affected the entire head, suggesting a diagnosis of tension headache. The patient was discharged from the intensive care unit after 10 days. Despite several drug trials, the headaches persisted. The same was true for cognitive impairment, despite cognitive rehabilitation with weekly speech therapy sessions. The patient was unable to resume his professional activity as a telephone network administrator at the national level. He was on occupational disability, living at home with a very good family environment.

**Figure 1:**
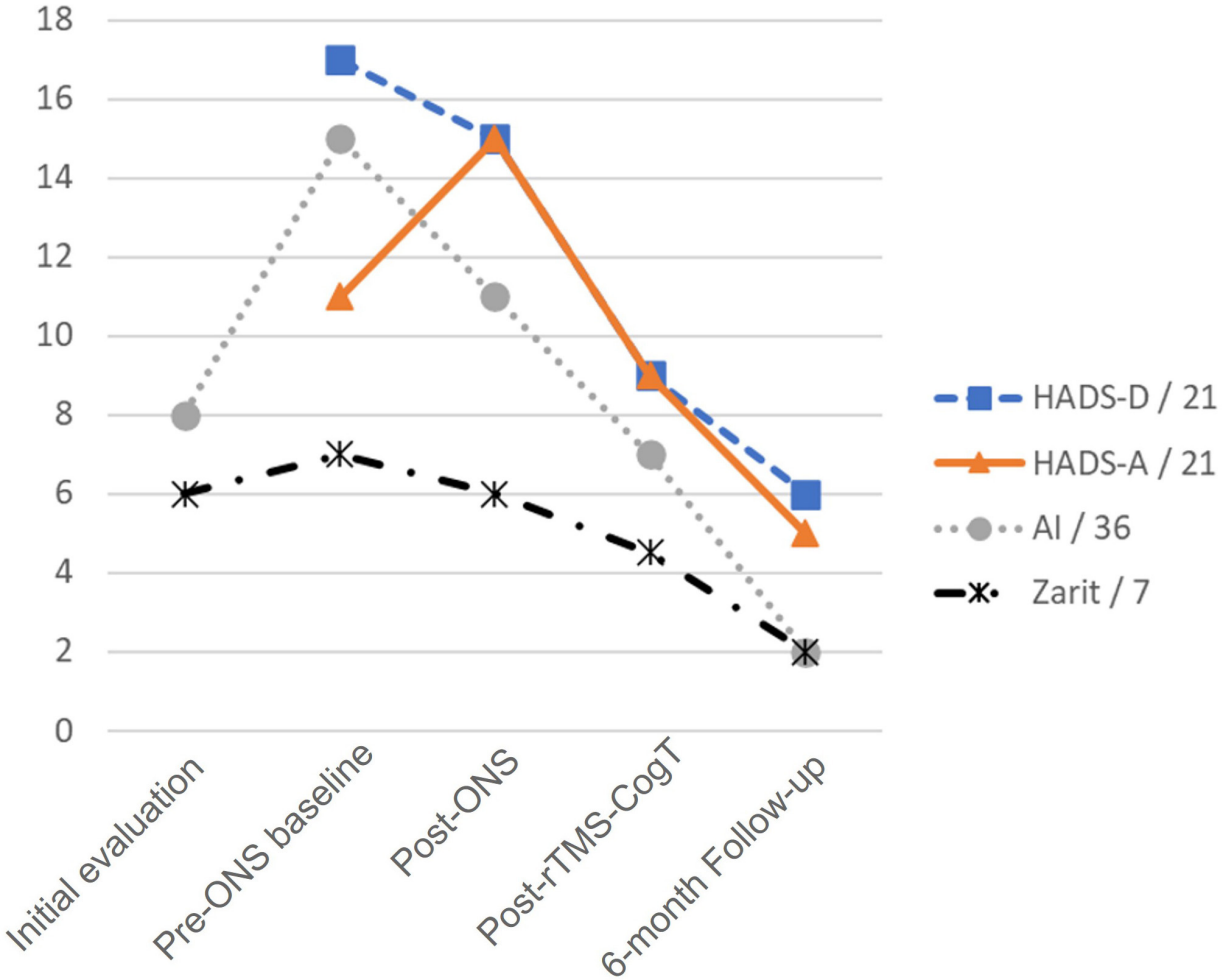
Evolution of clinical scores over time. Evolution of the Hospital Anxiety and Depression scale Depression (HADS-D) and Anxiety (HADS-A) subscores, the Apathy Inventory (AI) and the Zarit scores, at the following timepoints: (i) after 18 months of transcutaneous electrical neurostimulation of the occipital nerves: Initial evaluation; (ii) before bilateral implantation of occipital nerve stimulation (ONS): Pre-ONS baseline; (iii) 18 months after ONS implantation: Post-ONS; (iv) at the end of a subsequent 6-week therapy combining multisite repetitive transcranial magnetic stimulation and computerized cognitive training (rTMS-CogT): Post-rTMS-CogT; (v) 6 months after the end of the rTMS-CogT protocol: 6-month Follow-up.

He was referred to our center at the beginning of 2018 mainly due to permanent headaches that had become increasingly debilitating. A treatment with non-invasive transcutaneous electrical neurostimulation (TENS) applied to the both occipital nerves was initiated in February 2018 and proved to be remarkably effective. In July 2019, the neuropsychological assessment (initial evaluation) reported a total score of 19/30 on the Mini-Mental State Examination (MMSE) (23) (Table 1; Figure 1). Subsequently, the headaches gradually worsened as the patient used TENS less and less, which he considered too restrictive in daily life. At the same time, cognitive problems also increased, suggesting a link between the two symptoms. This led us to decide to implant an ONS device. The preoperative cognitive assessment performed at the end of May 2020 (pre-ONS baseline) confirmed the worsening of cognitive problems with a MMSE total score of 14/30. Cognitive disorders consisted of impaired executive functions, with a total score of 9/18 on the Frontal Assessment Battery (FAB) (24) (Table 1). In addition, psychomotor slowing was observed during visual attention and word reading tests. The patient also had great difficulty concentrating due to headaches.

**Table 1:** Clinical assessment performed initially after 18 months of transcutaneous electrical neurostimulation of the occipital nerves (ON-TENS), before bilateral implantation of occipital nerve stimulation (ONS), 18 months after ONS implantation, at the end of a subsequent 6-week therapy combining multisite repetitive transcranial magnetic stimulation and computerized cognitive training (rTMS-CogT), and finally 6 months after the end of the rTMS-CogT protocol.

	**Initial evaluation after 18 months of ON-TENS (July 2019)**	**Pre-ONS baseline (May 2020)**	**18-month follow-up post-ONS (pre-rTMS-CogT baseline) (March 2022)**	**Immediate evaluation after rTMS-CogT protocol (May 2022)**	**6-month follow- up after rTMS-CogT protocol (November 2022)**
Mini-mental state examination (MMSE) score/30	19	14	20	23	23
1. Orientation score/10	5	5	6	9	9
2. Registration score/3	3	3	3	3	3
3. Attention and calculation score/5	5	1	4	4	4
4. Recall score/3	0	0	0	0	0
5. Language score/8	5	4	6	6	6
6. Copying score/1	1	1	1	1	1
Frontal assessment battery (FAB) score/18	11	9	12	12	11
Phonemic fluency score/15^*^	11	11	13	14	11
Semantic fluency score/22^*^	5	4	4	7	8
Hospital anxiety and depression scale (HADS) anxiety subscore/21	-	11	15	9	5
Hospital anxiety and depression scale (HADS) depression subscore/21	-	17	15	9	6
Apathy inventory (AI) score/36	8	15	11	7	2
Disability assessment for dementia (DAD) score/100%	55	55	82.5	70	86.5
Zarit score/7	6	7	6	4.5	2
Alzheimer's disease assessment scale-cognitive subscale (ADASCog) score/70			24.75	18.05	18
1. Spoken language ability score/5			1	0	0
2. Comprehension score/5			1	0	0
3. Word finding difficulty score/5			3	1	1
4. Word recall task score/10			8	8.3	9
5. Naming objects and fingers score/5			2	0	1
6. Orientation score/8			5	2	1
7. Commands score/5			2	3	2
8. Ideational praxis score/5			0	1	0
9. Constructional praxis score/5			0	0	0
10. Word recognition task score/12			2.75	2.75	4
11. Remembering test instructions score/5			0	0	0

^*^Mean number of correct words recorded during 1 min in a healthy population (25, 26).

The surgical implantation of the ONS device (electrodes and pulse generator) was performed in September 2020. The continuous stimulation of the occipital nerves allowed headaches to be relieved very efficaciously. Additionally, cognitive performance gradually improved over a period of time and then stabilized after 1 year. In March 2022 (18 months after ONS implantation), the MMSE total score was 20/30, corresponding to a 43% improvement from pre-ONS implantation baseline, including increased attention and ability to perform calculations as well as in language functions (Table 1). At the same time, the FAB score was 12/18, corresponding to a 33% improvement of executive functions from pre-ONS implantation baseline. This resulted in greater autonomy [Disability Assessment for Dementia (DAD) (27) score: 82.5 vs. 55% at baseline, 33% improvement], but with only a slightly lower load for caregivers [Zarit score (28): 6/7 vs. 7/7 at baseline].

As the improvement produced by ONS did not seem sufficient and no longer progressing, we decided to perform an additional therapeutic approach, based on rTMS applied to multiple cortical sites combined with CogT. Such a combined protocol has been developed under the name NeuroAD^®^ therapy for the treatment of cognitive disorders, mainly apathy in the context of Alzheimer's disease (AD) (20–22). The detailed protocol we applied, similar to that used for patients with AD, has been described elsewhere (22). Briefly, this consisted of a daily session of rTMS-CogT for 5 consecutive days per week for a period of 6 weeks (30 sessions in total). Regarding rTMS, six different cortical regions were targeted, identified by a neuronavigation system (NeuroAD, Neuronix Ltd., Yoqnea'm, Israel) on the patient's brain magnetic resonance imaging (MRI): the right and left dorsolateral prefrontal cortices (DLPFC), the Broca's and Wernicke's language areas, and the right and left posterior parietal areas. On each region, 20 trains of 20 rTMS pulses were delivered at 10 Hz (2-s train duration and 40-s intertrain interval) for a total of 400 pulses over a period of 14 min. The intensity of stimulation was set at 100% of the rest motor threshold. During each intertrain interval of 40 s of rTMS interruption, the patient was asked to perform a cognitive task corresponding to the function of the stimulated cortical area: (i) naming of actions or objects, word recall, or spatial memory tasks for the DLPFC; (ii) syntax or grammar tasks for language areas; (iii) visuospatial attention tasks for parietal areas. Each task had six levels of gradual difficulty and the patients were allowed to progress to the next level of difficulty based on their performance in the previous session. During each daily session, three different cortical regions were treated by combining rTMS and corresponding CogT and the three other regions were treated the following day. Overall, each daily rTMS-CogT session lasted ~1 h.

The rTMS-CogT protocol was initiated in March 2022 and was completed without any adverse event or side effect. An initial assessment was performed just at the end of the 6 weeks of treatment (Table 1; Figure 1), including the Alzheimer's Disease Assessment Scale-Cognitive Subscale (ADASCog) (29) score. Unlike the MMSE, a lower ADASCog score reveals a better cognitive level. Following rTMS-CogT, the ADASCog score decreased by almost 7 points (27% improvement from pre-rTMS-CogT assessment), mainly in the naming objects, word finding, and orientation subscores. The orientation subscore of the MMSE also improved (from 6/10 to 9/10), leading to a 3-point increase in total MMSE score, as well as in semantic verbal fluency score. The rTMS-CogT therapy also produced beneficial effects on apathy [Apathy Inventory (AI) (30) score: 7/36 vs. 11/36 before rTMS-CogT, 36% improvement] and on depression and anxiety [Hospital Anxiety and Depression scale (HADS) (31) total score: 18/42 vs. 30/42 before rTMS-CogT, 40% improvement]. Finally, the caregiver burden was reduced (Zarit score: 4.5/7 vs. 6/7 before rTMS-CogT).

A follow-up assessment was performed 6 months after the end of the rTMS-CogT protocol (Table 1). At this time point, apathy, as well as depression and anxiety, further improved compared to the assessment performed just after the rTMS-CogT protocol (AI score: 2/36 vs. 7/36, 71% improvement; HADS total score: 11/42 vs. 18/42, 39% improvement). The caregiver burden was also greatly reduced (Zarit score: 2/7 vs. 4.5/7).

## Discussion

In this observation, a significant and lasting improvement in cognition and mood was achieved in a patient with a severe TBI after a sequence of two neuromodulation treatments including (1) bilateral implantation of ONS and (2) multisite rTMS performed in combination with CogT. The latter approach is generally dedicated to the treatment of cognitive disorders associated with AD (20–22). Our study is, to our knowledge, the first to have used this procedure to treat cognitive impairment secondary to head trauma. This therapeutic solution was justified in this patient who presented with memory, language and orientation disorders, such as those encountered in AD (32).

Cognitive disorders secondary to head trauma are related to complex biochemical processes, resulting in particular from damage to the blood-brain barrier in the white matter, later responsible for diffuse axonal damage (13–15). Depending on whether these lesions are located at the superficial cortical, subcortical or deep brain level close to the basal ganglia, quite different clinical sequelae can result. Superficial lesions are more likely to disrupt the functioning of different cortical areas and the connections between them, resulting in a picture of cognitive impairment similar to that observed in AD. Deep lesions are more responsible for disorders of consciousness, alertness and attention that are encountered in more or less severe vegetative states. In the case presented here, brain imaging showed that there was a left temporal impact, marked by the fracture of the temporal bone and its petrous part, and also lesions on the opposite side, marked by an acute subdural hematoma and a right temporoparietal contusion. These findings suggest traumatic lesions secondary to a rotation mechanism in the coronal plane (33) theoretically responsible for axonal lesions of moderate severity but affecting both the subcortical and deep brain regions (34).

However, cognitive impairment following head trauma is not solely determined by white matter lesions. The homeostatic balance between inhibition and excitation is also disrupted in the brain's neural networks following TBI (35). Gamma-aminobutyric acid (GABA) is the major inhibitory neurotransmitter and glutamate the major excitatory neurotransmitter in the central nervous system. There is evidence for the occurrence of an immediate rise in glutamate levels following severe TBI in humans (36). A disruption in GABAergic signaling may lead to a further increase in glutamate excitotoxicity, which can worsen the impact of neuronal damage (37). By being able to modulate the GABA/glutamate balance and producing long-lasting effects on synaptic transmission (38–40), non-invasive brain stimulation techniques, such as rTMS, have a certain interest in this clinical context (41). However, it is difficult to have preconceptions regarding the type of rTMS pattern to apply to modulate the GABA/glutamate balance after TBI, particularly according to the influence of metaplasticity processes (42). Indeed, while rTMS tonically applied at low frequency ( ≤ 1 Hz) is known to be able to depress long-term synaptic transmission and is therefore potentially neuroprotective, high-frequency rTMS (as applied in our patient), although considered excitatory, has also shown neuroprotective or pro-GABAergic effects in various experimental models (43–45) or clinical conditions (46–48). Furthermore, the situation is more complex than a dual mechanism of increased vs. decreased excitability, because cognitive recovery after TBI depends on various neural repair processes, including restoration and synchronization of neuronal network connectivity for cognitive performance, in which the modulation of tonic and phasic GABA levels plays a complex interaction role.

Our results show that combined rTMS-CogT therapy may be a well-suited approach to promote post-TBI cognitive recovery. However, performing such a protocol requires good attentional and psychomotor capacities to complete the cognitive tasks quickly within the time imposed by the rTMS protocol. Our patient initially presented with great difficulty concentrating, mainly due to disabling headaches, which could have initially prevented him from complying with the rTMS-CogT protocol. This is the reason why it appeared to us that the priority was to treat his headaches first, which led us to propose the treatment by ONS. High analgesic efficacy of this neuromodulation technique has been reported in various types of non-migrainous chronic headaches (49) and was therefore confirmed in our patient. To our knowledge, implanted ONS has never been proposed before to relieve refractory headaches secondary to TBI, but clearly appears to be an interesting therapeutic solution in this context.

The ONS probably made it possible to take a first step in cognitive improvement in our patient, but indirectly, thanks to the reduction of pain. However, a cognitive improvement more directly produced by ONS was also possible, as suggested by a previous study showing the increase in memory performance thanks to the application of tDCS to stimulate the greater occipital nerve (50), possibly via the activation of the locus coeruleus (51). Other authors have suggested that ONS may also improve attention by acting on the thalamus or basal ganglia (52, 53). Thus, our patient improved his attentional and executive functions following ONS therapy, but this proved insufficient to restore quality daily life and the rTMS-CogT protocol seemed to us to be a good approach to further improve the cognitive performance and reduce mood disorders to a clinically satisfactory level.

Various rTMS protocols have already been proposed to treat the clinical symptoms associated with concussion (mild TBI) or more severe TBI and this has been the subject of about 40 publications since 2006, including three recent reviews (54–56) and three meta-analyses (57–59). Symptoms intended to be treated by rTMS, primarily targeted to the left DLPFC at high frequency (or more rarely to the right DLPFC at low frequency), were disorders of consciousness, dizziness, auditory disorders, motor dysfunction, pain, headache, depression, or cognitive impairment, including post-concussion syndrome after mild TBI.

All recent reviews and meta-analyses (54–59) have concluded that there is significant evidence for the efficacy of rTMS of the DLPFC as a therapeutic intervention for depression, headache or pain associated with TBI. In contrast, the effects were more moderate and variable with respect to the improvement of cognitive performance, including executive functions, attentional abilities, and memory, except perhaps for visuospatial memory tasks, whereas the level of evidence was very low for disorders of consciousness.

For example, in a series of 21 patients with refractory post-TBI headache, 4 sessions of 10 Hz rTMS administered to the left DLPFC showed a small but significant improvement in depressive symptoms on the Hamilton Rating Scale for Depression score (3-point reduction, 15% from baseline) after active but not sham rTMS, beyond major analgesic effects on headaches (60). However, other studies have shown less significant effects of rTMS on depression associated with TBI. First, in a series of 30 patients, only small and very variable beneficial effects were observed on depression following a protocol of 20 sessions of 1 Hz rTMS delivered to the right DLPFC (61). Second, in a series of 21 patients, no differences were observed between active and sham protocols of 20 sessions of sequential bilateral rTMS to the right and left DLPFC (62). Furthermore, in these studies, rTMS therapy improved post-concussion subjective symptoms (61) or cognitive performance regarding executive functions and working memory (62).

Thus, given the results of these previous studies, one can question the relevance of delivering rTMS at low (1 Hz) or high (10–20 Hz) frequency of stimulation to the DLPFC target depending on its laterality (right or left) and also the method to determine the optimal location of this DLPFC target (using cranial landmarks or image-guided navigation). On the one hand, in our case, rTMS trains were applied at high frequency on the DLPFC, whatever the hemisphere. On the other hand, one group targeted the DLPFC with individualized resting-state network brain mapping of the functional connectivity between the subgenual anterior cingulate cortex (sgACC) and the default-mode network using functional magnetic resonance imaging (63, 64). After 20 sequential bilateral rTMS sessions on this individualized target (low-frequency stimulation on the right side and high-frequency stimulation on the left side), TBI-associated depression was improved twice by active stimulation than by sham stimulation on the Montgomery-Asberg Depression Rating Scale score.

However, beyond depression, our study mainly showed rTMS-induced improvement on various cognitive symptoms. In the literature, at least six studies have evaluated the effects of rTMS delivered at high frequency over the left DLPFC on cognitive impairment associated with TBI (65–70). First, in 12 patients with mild TBI, 20 sessions of 10 Hz-rTMS delivered to the left DLPFC improved post-concussion symptoms, including cognitive deficits (mainly memory disturbances), for <3 months (65). Second, in 26 patients with cognitive complaints and a history of mild-to-moderate TBI, 5 sessions of 10 Hz-rTMS delivered to the left DLPFC improved executive functions and subjective measures of cognitive dysfunction related to a post-concussion syndrome, up to 2-week follow-up (66). In contrast, no effect of treatment was observed on cognitive test performance assessing selective attention control and verbal learning or fluency. Third, in 18 patients with persistent post-concussion syndrome, 13 sessions of 20 Hz rTMS delivered to the left DLPFC produced significant cognitive improvement up to 2 months after the intervention, but only in patients with recent TBI (<12 months) (67). In contrast, our patient benefited from rTMS therapy 10 years after the initial trauma. Finally, in two studies of patients with TBI, the overall effect of 10 Hz rTMS delivered to the left DLPFC produced either cognitive improvement below clinically meaningful thresholds (69) or no significant changes in executive function evaluated using the Trail Making Test Part B or other neuropsychological tests for attention, learning and visuospatial memory (70).

Concerning low-frequency (1 Hz) rTMS delivered to the right DLPFC, a protocol of 30 sessions twice-daily applied to 15 patients with mild TBI showed significant improvement in different post-concussion symptoms, such as pain, depression and anxiety, as well as in cognitive tasks assessing verbal fluency, working memory, selective attention, and cognitive processing speed (71). In contrast, there were no significant changes in executive functioning, fatigue severity, or apathy.

In all these studies, rTMS was applied in isolation, mainly targeting the left DLPFC and not associated with CogT at the same time. Only one study has previously evaluated the benefit of treating TBI-associated cognitive impairment by combining rTMS and CogT (72). In this retrospective study of 166 patients, half received rTMS and CogT and the other half (control group) various usual methods of treatment (72). The protocol was not well described but was based on 1 Hz-rTMS delivered to the DLPFC (likely to the right hemisphere) once a day, 5 days a week for 3 months, in combination with CogT (but not performed during the rTMS protocol), including various tasks to improve concentration, visuospatial memory, visual perception, judgment and reasoning. Cognitive improvement was significantly better in the rTMS-CogT group than in the control group.

Our case highlights several original elements likely to improve the therapeutic management of patients with TBI.

First, the benefit of using ONS to treat chronic refractory headaches secondary to TBI, even several years after the head trauma, must be emphasized, as this is the first case reported here. Our case broadens the spectrum of indications for this technique. It is important to point out that it is possible to perform this neuromodulation strategy non-invasively, using a TENS technique. ONS-TENS can be a temporary solution, but if this technique is effective but insufficient to control headaches over time, then this efficacy can be predictive of a good outcome provided by implanted ONS (73, 74).

Second, our case shows that the implantation of ONS can be well tolerated in patients who have previously had a head trauma, and that this implanted neuromodulation technique does not prohibit the subsequent performance of rTMS sessions, even on different cortical sites such as the posterior parietal areas.

Third, our case also shows that the treatment of headaches associated with TBI is a therapeutic priority, making it possible to trigger a virtuous circle of management of other post-concussion symptoms. Perhaps also the ONS could have direct beneficial effects on certain central dysfunctions, for example in the cognitive domain.

Fourth, the clinical results obtained in this patient may also suggest that the prior ONS could have set the stage for a significant and lasting improvement in cognitive performance and mood produced by the subsequent rTMS-CogT protocol. Persistent cognitive disorders may be responsible for anxiety and depression (75), possibly by alteration of dopaminergic circuits in the context of TBI, mainly concerning the striatum and the frontal/prefrontal cortex (76–78). It is therefore conceivable that the combination of techniques capable of modulating deep brain structures such as the basal ganglia on the one hand (ONS) and the cortical brain networks on the other hand (rTMS) could have a synergistic interest in reactivating dopaminergic circuits in order to improve various post-concussion symptoms in the context of TBI. This hypothesis could be tested in the future, in particular by functional brain imaging techniques.

Finally, our results support a likely greater efficacy (in terms of magnitude and duration) on improving cognitive performance and mood by means of a combined treatment with multisite rTMS and CogT compared to the rTMS strategy usually applied in isolation in the context of TBI, which is the stimulation of the DLPFC only (usually at high frequency on the left hemisphere). However, it is difficult to distinguish between (i) a potential beneficial effect of multisite stimulation related to the total stimulation dose or the modulation of cerebral connectivity, (ii) the specific effect of CogT, or (iii) the possible synergy between both approaches.

Of course, a single case does not justify unqualified approval of the techniques used. Additionally, given that most clinical measures improved linearly over time, this may suggest that the entire treatment received regularly may have benefited the patient rather than a specific technique (ONS or rTMS-CogT). It is also clear as we have repeatedly pointed out that the remission of the headaches contributed greatly to the overall improvement of the patient. Finally, it is obvious that this case does not eliminate a placebo effect of the different neuromodulation techniques used, and that the simple fact of being included in an innovative therapeutic program could have improved his symptoms, regarding mood for example. It must, however, be emphasized that the beneficial therapeutic effects were obtained very far from the initial traumatic episode and over a prolonged period of several years, which supports the real efficacy of the neuromodulation techniques that were added. In any case, this original observation opens the prospect of a controlled study on a larger sample, evaluating the effects on the various post-concussion symptoms that can be produced by means of an active multisite rTMS protocol compared to a sham condition and associated or not with a CogT protocol.

## Data availability statement

The original contributions presented in the study are included in the article/supplementary material, further inquiries can be directed to the corresponding authors.

## Ethics statement

Written informed consent was obtained from the individual(s) for the publication of any potentially identifiable images or data included in this article.

## Author contributions

TC and J-PN: conceived and designed the study. J-PL and AS: provided supervision. EL, SL, and SD: collected the data. All authors contributed to manuscript revision and read and approved the final version.
